# Consumer Perspectives on the Barriers to Timely Palliative Care Access: A Mixed Methods Study

**DOI:** 10.1111/jocn.70480

**Published:** 2026-08-05

**Authors:** Alannah L. Cooper, Dipna Martin‐Robins, Rosemary Dillon, John Clements, Huaqiong Zhou, Janie A. Brown

**Affiliations:** ^1^ School of Nursing, College of Health and Education Murdoch University Murdoch Western Australia Australia; ^2^ St John of God Subiaco Hospital Subiaco Western Australia Australia; ^3^ Palliative Care Australia Deakin Australian Capital Territory Australia; ^4^ School of Nursing Curtin University Bentley Western Australia Australia; ^5^ St John of God Midland Public and Private Hospital Midland Western Australia Australia; ^6^ The Western Australian Group for Evidence Informed Healthcare Practice Curtin University, Perth Western Australia Australia

## Abstract

**Aim:**

To identify the barriers to timely access to palliative care from the perspective of Australian inpatients and their families.

**Design:**

A co‐designed mixed methods study with an equal status explanatory design (QUAN ➔ QUAL).

**Methods:**

Quantitative data were collected in Phase 1 via surveys to assess participants' knowledge and experience of palliative care. In the second phase, qualitative data were obtained via semi‐structured interviews to explore participants' perceptions of palliative care and to identify barriers to palliative care.

**Results:**

A total of 194 complete patient survey responses and 52 family/visitor responses were received. Mean self‐rated knowledge of palliative care was slightly higher than self‐rated knowledge of end‐of‐life care in both groups. Patient respondents scored a mean of 9.3 out of 13 on the Palliative Care Knowledge Scale and family visitor respondents had a mean score of 9.7. Statistically significant differences in the mean Palliative Care Knowledge scores were found when patient and family respondents reported being familiar with palliative care. A total of 11 patients and nine family members participated in an interview in Phase 2. Three main themes and nine subthemes were identified. The three main barriers were Communication, Knowledge and Perceptions, and Accessibility.

**Conclusion:**

Patients and family members perceived a range of barriers to palliative care at the individual, health professional, and system level. These findings highlight areas where targeted interventions should be developed to improve timely access to palliative care.

**Impact:**

- While the perspectives of health professionals are well known, the perceptions of patients and families on the barriers to palliative care are underreported.
- A wide variety of complex issues that could impede timely access to palliative care were identified at the individual, health professional and system level.
- Health consumer perspectives are essential to fully understanding the barriers to palliative care access. The findings of this research can be used to inform future interventions to improve access to palliative care.

**Reporting Method:**

Good Reporting of A Mixed Methods Study (GRAMMS) checklist (Data S2) (O’Cathain et al., 2008).

**Patient or Public Contribution:**

The study was guided by a steering group of health consumers and health professionals working in partnership with the research team through all stages of the research process.

## What Does This Paper Contribute to the Wider Global Clinical Community?

- Insights on the barriers to palliative care from the perspective of health consumers
- Consumer perspectives that provide a fuller understanding of the complex issues that impede palliative care access
- Consumer perspectives that will inform interventions to improve timely palliative care access

## 1 Introduction

Palliative care is a holistic approach to healthcare that addresses the physical, social, psychological, and spiritual needs of people with life‐limiting illnesses (World Health Organisation 2024). The World Health Organisation (WHO) considers palliative care to be an essential element of holistic healthcare and has encouraged global action to improve access (WHO 2020, 2024). However, despite advocacy from the WHO and other leaders in the field, significant barriers remain (Thomas et al. 2022; WHO 2024). Global estimates indicate only 14% of people who require palliative care receive it (WHO 2020). Similarly, research studies investigating inpatient hospital palliative care need have found high levels of unmet need, with 61%–71% of patients with potential need not receiving any form of palliative care (Cooper et al. 2025, 2022; Szekendi et al. 2016). The gap between need and access is expected to continue to worsen with an ageing population and increasing onset of non‐communicable diseases (Palliative Care Australia 2021; WHO 2020).

The provision of palliative care can occur in a specialist or generalist capacity (Gouldthorpe et al. 2023). Generalist palliative care can be provided by all health professionals and supports patients with less complex care needs, such as basic pain control, supporting a patient's independence, and offering support to patients and their families during illness and death (Palliative Care Australia 2018). Specialist palliative care is provided by healthcare professionals with extensive training and experience in the specialty and is primarily for patients with complex needs and symptoms that remain poorly controlled despite generalist palliative care approaches (Gouldthorpe et al. 2023; Palliative Care Australia 2018).

### 1.1 Background

Timely palliative care is defined as palliative care provided at least 3 months before death (Mah et al. 2024). Early implementation through the provision of generalist palliative care and referral to specialist palliative care when appropriate improves symptom control, maximises quality of life, and reduces healthcare costs (Janberidze et al. 2021; Mah et al. 2024; Srinivasan et al. 2023). Despite the known benefits of timely palliative care, this is poorly implemented in practice (Cooper et al. 2025; Tenge et al. 2024; Willinger et al. 2023). The median duration of specialist palliative care across 23 countries included in a meta‐analysis was 18.9 days (Jordan et al. 2020). Australia had the lowest median of 6 days. More recent national data compiled by the Australian Institute of Health and Welfare (AIHW) (2024a) reported a median duration of 15 days of specialist palliative care prior to death still falling well short of the optimum duration. The data internationally and locally highlight significant delays in accessing palliative care.

Identifying the barriers to palliative care is crucial to addressing the high levels of unmet need currently reported (Cooper et al. 2022; WHO 2020). A recent qualitative meta‐synthesis identified a range of barriers including misconceptions of palliative care from patients, caregivers and clinicians, a lack of trust and poor communication between healthcare professionals, a lack of time to provide palliative care, and sociodemographic factors such as income, culture, and health literacy (Slusser et al. 2024). Insufficient staffing has also been identified as a barrier to palliative care provision (Klaiman et al. 2024; Rost et al. 2020). Additionally, while all healthcare professionals should be able to provide generalist palliative care, there is an apparent lack of education and training to enable this practice (Klaiman et al. 2024; WHO 2024). As a result, specialist palliative care services are required to take on a greater patient burden despite limited resources to do so.

While there is a growing understanding of the barriers to palliative care, limitations remain (Slusser et al. 2024; WHO 2020). Many studies report on the perspectives of doctors, nurses, and other healthcare professionals, with little or no input from healthcare consumers (de Barbieri et al. 2024; Jones et al. 2023; Zhang et al. 2024). As a result, the perceptions of patients and families are underreported. Additionally, most of the research investigating barriers to palliative care has been conducted in Europe and America (de Barbieri et al. 2024; Slusser et al. 2024). Exploration of the barriers to palliative care within the Australian context and from the perspective of health consumers is required to broaden understanding and facilitate the development of effective interventions to improve access.

## 2 The Study

### 2.1 Aim and Objectives

The aim of this study was to identify the barriers to timely access to palliative care from the perspective of Australian inpatients and their families. The objectives of the study were to:

1. Explore perceptions of palliative care from patient and carer/family/visitor perspectives,
2. Identify barriers to generalist palliative care approaches and specialist palliative care referral,
3. Understand the barriers to generalist palliative care approaches and specialist palliative care referral.

The Good Reporting of A Mixed Methods Study (GRAMMS) checklist (Data S2) (O'Cathain et al. 2008) guided the reporting of the study.

### 2.2 Design

A co‐designed mixed methods study with an equal status explanatory design (QUAN ➔ QUAL) (Ivankova et al. 2006; Tashakkori and Teddlie 2010). Adopting a co‐design approach actively engaged end‐users in the research process (Jessup et al. 2018). The study was guided by a steering group of consumers and health professionals working in partnership with the research team on all aspects of the research (Figure 1). Integration took place at the method, interpretation, and reporting level (Fetters et al. 2013). At the method level, integration quantitative data connected to the qualitative data through the sampling frame; participants who completed Phase 1 were invited to take part in Phase 2. Building was also used, with the interview guides for Phase 2 developed with reference to the Phase 1 quantitative results. Finally, integration took place at the interpretation and reporting level where quantitative results and qualitative findings were drawn together.

**FIGURE 1:**
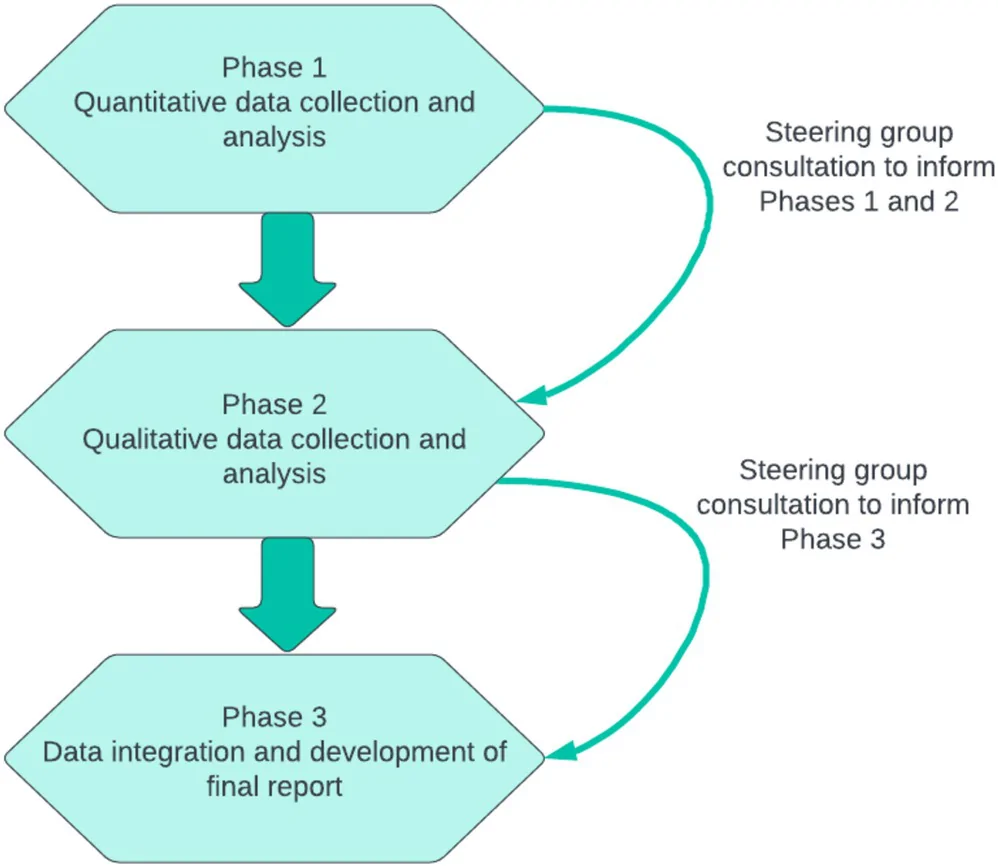
Study design and phases. [Colour figure can be viewed at wileyonlinelibrary.com]

### 2.3 Phase 1: Quantitative Methods

#### 2.3.1 Study Setting and Sampling

The study site was a private, not‐for‐profit, metropolitan hospital in Western Australia with 578 beds. At the time of the study, the hospital offered a range of medical and surgical services, caring for inpatients, outpatients and day patients.

#### 2.3.2 Inclusion and Exclusion Criteria

##### 2.3.2.1 Patients

The survey was open to all adult patients who received care at the study site during the survey period. Patients under 18 years of age and patients who did not have capacity to consent met the exclusion criteria for the survey.

##### 2.3.2.2 Family

The survey was open to all carers/family members visiting or accompanying an adult patient who was receiving any type of care at the study site during the survey period. Family included carers, spouses, friends, and traditional family member relations and are referred to collectively as family from this point onwards. Family who were visiting/accompanying a patient under 18 years of age met the exclusion criteria for the survey.

#### 2.3.3 Data Collection

Data were collected via cross‐sectional surveys. Patients and family members were invited by the research team to complete questionnaires electronically or by paper. The surveys for each group were open from 23rd of October 2023 to 27th December 2023. Data were collected on respondents' characteristics and two validated tools were used to assess patient and family respondents' knowledge and experience of palliative care. A third validated instrument was administered to patient respondents to assess how they would feel if a palliative care approach was integrated into their care. Patient respondents were also asked if they had a history of any of the 12 conditions known to benefit from palliative care outlined in the Gold Standards Framework Proactive Identification Guidance (GSF PIG) (Thomas and Armstrong Wilson 2016). The final item in the questionnaire asked all respondents if they would be interested in taking part in an interview for the second phase of the study.

##### 2.3.3.1 Palliative Care Knowledge Scale (PaCKS)

The PaCKS is a validated 13‐item scale that assesses an individual's knowledge of palliative care (Kozlov et al. 2017). The scale consists of 13 statements and participants are asked to select if they think the statement is true, false, or if they don't know if the statement is true or false (Kozlov et al. 2017, 2018). The total possible score is 13 with a higher score indicating a higher level of knowledge of palliative care. A score of one point is given for correct answers and incorrect or don't know answers score zero points.

##### 2.3.3.2 Experience and Familiarity With Palliative Care Questionnaire

This validated 8‐item questionnaire is designed to be used alongside PaCKS and provides adjunct information about participants' self‐rated experience, familiarity, and knowledge of palliative care (Kozlov et al. 2017). Level of agreement (strongly agree—strongly disagree) with statements relating to experience and familiarity were measured via 5‐point Likert scales. Self‐rated knowledge was scored from 0 to 10 with a score of 0 indicating ‘I know nothing at all’ and a score of 10 ‘I am very knowledgeable’ (Kozlov et al. 2017, 532). Minor amendments were made to terminology to reflect the services available at the study site for two questions with the term ‘hospice care’ changed to ‘end‐of‐life care’.

##### 2.3.3.3 Perceptions of Palliative Care Questionnaire

Four questions from the perceptions of palliative care questionnaire (Chosich et al. 2020) were used to measure patient respondents' feelings about palliative care. Respondents completing the patient questionnaire were asked to rate their level of agreement (strongly agree—strongly disagree) on how they would feel if palliative care became involved in their care.

#### 2.3.4 Data Analysis

Statistical analysis was performed using the statistical software program Stata 16 (StataCorp 2019). Descriptive statistics including frequencies and percentages were used to describe the participant characteristics for each group. Comparisons within and between groups were made using inferential statistics. The distributions for each variable were tested and parametric or non‐parametric tests were applied as appropriate. For example, mean levels for PaCKS scores were normally distributed so a *t*‐test was performed (Sainani 2012) to compare patient and family groups. For abnormally distributed variables, the Wilcoxon rank‐sum test or the Kruskal‐Wallis H test were used as appropriate (Sainani 2012). For example, to compare PaCKS scores and the self‐rated knowledge about palliative care, the Kruskal‐Wallis H test was performed.

### 2.4 Phase 2: Qualitative Methods

#### 2.4.1 Study Setting and Sampling

Purposive sampling was used to select participants from those that had indicated interest in taking part in an interview for Phase 2. The research team reviewed the respondents' characteristics, PaCKS and experience and familiarity scores to select participants with varying levels of knowledge and experience, and to include participants from different age groups, genders, and with attendance to hospital for a variety of conditions. Given the narrow focus of the study at a single site, use of purposive sampling, and the depth of data anticipated from semi‐structured interviews, 8–10 participants per group were expected to provide sufficient information power (Braun and Clarke 2022; Malterud et al. 2016).

#### 2.4.2 Inclusion and Exclusion Criteria

Patient or family respondents who had participated in Phase 1 of the study and had indicated they would be willing to participate in Phase 2 were eligible.

#### 2.4.3 Data Collection

Prior to commencing qualitative data collection, Phase 1 quantitative results were presented to the steering group to assess patterns or areas of interest to explore in Phase 2. Potential questions developed at the conception of the project were reviewed and interview guides for the patient and family participant groups were refined and finalised, drawing on the Phase 1 results (Figure 1). Individual, one‐on‐one, semi‐structured interviews were conducted at a time and place or medium (face‐to‐face, phone or video call) that was convenient for participants. Open‐ended questions were used to allow participants to provide deep and detailed answers, and so the interviewer could clarify and delve further during this process to gain maximum data from the interviews (Turner 2010). The interviews were conducted by a Nurse Researcher (AC) and Research Nurse (DMR) who had no prior relationship with the participants and had relevant research and clinical knowledge. The interviews were audio recorded and transcribed verbatim.

#### 2.4.4 Data Analysis

Reflexive thematic analysis was undertaken following the six steps developed by Braun and Clarke (2006) outlined in Table 1. NVivo (QSR‐International 2015) was used to assist in the analysis process.

**TABLE 1:** Qualitative analysis process.

Steps of analysis (Braun and Clarke 2006)	Action
Step 1: Familiarising yourself with the data	Research team conducted the interviews Audio recordings were listened to twice prior to transcription Transcripts read in entirety multiple times
Step 2: Generating initial codes	Entire data set was coded Coding was completed independently by two researchers (AC & DMR)
Step 3: Searching for themes	Assessed for similarity of concepts within codes Codes presented to the steering group Steering group members independently grouped codes into initial themes Initial themes developed were discussed in the steering group Initial thematic map developed
Step 4: Reviewing themes	Compared and contrasted initial themes for similarities Re‐coding Discussion of themes by researchers and steering group Development of thematic map Compared themes to transcripts Found quotes that conveyed themes and subthemes
Step 5: Defining and naming themes	Essence of each theme was identified Detailed analysis of each theme written Reflection of themes against study aims Scope of each theme was determined Theme names were finalised
Step 6: Producing the report	Findings were written up with supporting evidence of themes within the data

#### 2.4.5 Mixed Methods Integration

A joint display was used to facilitate side‐by‐side comparison of the quantitative results and qualitative findings and draw meta‐inferences (Haynes‐Brown and Fetters 2021). Data sources were examined for confirmation, expansion and discordance to assess the fit of integration (Fetters et al. 2013).

#### 2.4.6 Ethical Considerations

This study received ethical approval from the study hospital (ref:2120) and university human research ethics committees (HRE2023‐0610). Participants were provided with participant information sheets for Phase 1 and Phase 2 that outlined the purpose and nature of the study. Consent for the Phase 1 survey was inferred by completion and return of the questionnaire. Written consent was obtained by a Nurse Researcher (AC) and Research Nurse (DMR) prior to the Phase 2 interviews. Given the sensitive nature of palliative care, there was a risk that participating could result in distress for participants. All participants were provided with details about available support services they may wish to contact in the event they became distressed.

## 3 Results

### 3.1 Phase 1 Quantitative Results

#### 3.1.1 Characteristics of the Patient Sample

A total of 208 responses to the patient survey were received; 14 incomplete responses were excluded from analysis, leaving a final sample of *n* = 194. The characteristics of respondents that completed the patient survey are outlined in Table 2. The majority of respondents (60%, *n* = 115) reported experiencing one or more conditions listed in the GSF PIG (Thomas and Armstrong Wilson 2016).

**TABLE 2:** Patient survey respondent characteristics.

Variable	Sample (*n* = 194)
Age mean (range) SD	65 (24–97) 14.07
**Gender**	
Woman	52% (*n* = 101)
Man	48% (*n* = 93)
**Speciality**	
Bariatric	0.5% (*n* = 1)
Cardiology	4% (*n* = 8)
Cardiothoracics	2% (*n* = 3)
Colorectal	3% (*n* = 5)
Gastroenterology	0.5% (*n* = 1)
General surgery	2% (*n* = 4)
Geriatrics	0.5% (*n* = 1)
Gynaecology	3% (*n* = 5)
Haematology	1% (*n* = 2)
Infectious diseases	2% (*n* = 3)
Nephrology	0.5% (*n* = 1)
Neurology	1% (*n* = 2)
Neurosurgery	9% (*n* = 17)
Oncology	24% (*n* = 47)
Orthopaedics	20% (*n* = 38)
Pain	1% (*n* = 2)
Plastics	4% (*n* = 7)
Respiratory	3% (*n* = 5)
Spinal surgery	16% (*n* = 32)
Urology	4% (*n* = 7)
Unknown	2% (*n* = 3)
**History of GSF PIG condition**	
One or more of the conditions listed	60% (*n* = 115)
None of the conditions listed	40% (*n* = 79)
**Education**	
Advanced diploma/Diploma	8% (*n* = 15)
Bachelor's degree	17% (*n* = 32)
Certificate I/II/III/IV	16% (*n* = 31)
Graduate diploma/Graduate certificate	7% (*n* = 13)
High school	37% (*n* = 71)
Other	2% (*n* = 3)
Postgraduate degree	13% (*n* = 25)
Primary School	2% (*n* = 4)
**Employment**	
Between Jobs	2% (*n* = 3)
Casual employment	2% (*n* = 3)
Employed full time	23% (*n* = 44)
Employment part time	7% (13)
Homemaker	1% (*n* = 2)
Prefer not to answer	1% (*n* = 2)
Retired	51% (*n* = 98)
Self‐employment	7% (*n* = 13)
Student	1% (*n* = 2)
Unable to work	6% (*n* = 12)
Unemployed	1% (*n* = 2)
**Current or previous experience working in healthcare**	
Yes	25% (*n* = 49)
No	75% (*n* = 145)
**Provided carer support to family member or friend**	
Yes	26% (*n* = 51)
No	74% (*n* = 143)

#### 3.1.2 Patient Palliative Care Knowledge

Respondents' mean self‐rated knowledge of palliative care was slightly higher than mean self‐rated knowledge of end‐of‐life care. Comparisons of mean self‐rated knowledge and assessment of an individual's knowledge of palliative care through mean PaCKS scores are presented in Table 3.

**TABLE 3:** Comparison of self‐rated knowledge scores and PaCKS scores.

Measure	Patient respondents *n* = 194	Family respondents *n* = 47
Mean self‐rated knowledge of palliative care^a^ (SD)	4.7 (2.1)	5.3 (2.3)
Mean self‐rated knowledge of end‐of‐life care^a^ (SD)	4.3 (2.4)	4.8 (2.4)
Mean PaCKS score^b^ (SD)	9.3 (3.4)	9.7 (3.1)

^a^ Possible scores ranged from 1 to 10 with a higher score indicating a higher level of self‐rated knowledge.

^b^ Possible scores ranged from 0 to 13 with a higher score indicating higher knowledge of palliative care.

#### 3.1.3 Patient Experience and Familiarity With Palliative Care

The majority of the respondents had heard of palliative care (93%, *n* = 181) and end‐of‐life care (79%, *n* = 154) and also reported being familiar with palliative care (68%, *n* = 132) and end‐of‐life care (53%, *n* = 102). Self‐reported experience of palliative care (39%, *n* = 76) and end‐of‐life care (31%, *n* = 60) was low.

#### 3.1.4 Perceptions Regarding Palliative Care

The majority of respondents agreed that they would feel comforted if palliative care was involved in their treatment (68%, *n* = 133). Responses in relation to feeling frightened, feeling hopeless, and preferences for how palliative care is described were mixed (Table 4).

**TABLE 4:** Responses to perceptions regarding palliative care.

Item	Strongly agree	Agree	Neutral	Disagree	Strongly disagree
I would feel comforted if palliative care is involved in my treatment	30% (*n* = 59)	38% (*n* = 74)	23% (*n* = 45)	4% (*n* = 8)	4% (*n* = 8)
I would feel frightened if referred to palliative care	12% (*n* = 23)	27% (*n* = 53)	26% (*n* = 51)	24% (*n* = 46)	11% (*n* = 21)
I would feel hopeless if referred to palliative care	9% (*n* = 17)	15% (*n* = 30)	30% (*n* = 58)	32% (*n* = 62)	14% (*n* = 27)
I would feel more comfortable being referred to ‘supportive care’ services rather than ‘palliative care’ services	12% (*n* = 23)	28% (*n* = 55)	39% (*n* = 76)	16% (*n* = 32)	4% (*n* = 8)

#### 3.1.5 Within Group Comparisons

The mean PaCKS score was 9.3 (SD 3.4, range 1–13). There was a statistically significant difference in PaCKS scores between respondents who had experience working in healthcare compared to those who did not (*p* = 0.0005). There were no statistically significant differences for mean PaCKS scores based on gender (*p* = 0.0963), educational attainment (*p* = 0.3280), employment status (*p* = 0.4181) or providing carer support (*p* = 0.4968).

Comparisons were made between PaCKS scores and responses to items in the Experience and Familiarity with Palliative Care Questionnaire (Table 5). Statistically significant differences in the mean PaCKs score were found between all variables, indicating that respondents who had heard of, were familiar with, had experience of, or rated their knowledge of palliative care or end‐of‐life care had higher PaCKs scores.

**TABLE 5:** Comparison of patient respondents PaCKS scores and responses to items in the Experience and Familiarity with Palliative Care Questionnaire.

Experience and familiarity with palliative care		Median difference of PaCKS scores
Palliative care	Heard of	*z* = −3.857, *p* = 0.0001*
	Familiar with	*Χ* ^*2*^ (2) = 19.781, *p* = 0.0001**
	Experience with	*Χ* ^*2*^ (2) = 21.685, *p* = 0.0001
	Self‐ rated knowledge	*Χ* ^*2*^ (9) = 49.251, *p* = 0.0001
End of life care	Heard of	*z* = −4.420, *p* = 0.0000
	Familiar with	*Χ* ^*2*^ (2) = 32.855, *p* = 0.0001
	Experience with	*Χ* ^*2*^ (2) = 13.364, *p* = 0.0013
	Self‐ rated knowledge	*Χ* ^*2*^ (9) = 44.342, *p* = 0.0001

* Wilcoxon rank‐sum test.

** Kruskal‐Wallis Test.

#### 3.1.6 Characteristics of the Family Sample

A total of 52 family members responded to the survey. Five incomplete responses were excluded from analysis, leaving *n* = 47 responses for analysis. Family respondent characteristics are presented in Table 6.

**TABLE 6:** Family survey respondent characteristics.

Variable	Sample (*n* = 47)
Age mean (range) SD	60 (29–87) 14.81
**Gender**	
Woman	68% (*n* = 32)
Man	30%% (*n* = 14)
Other	2% (*n* = 1)
**Education level**	11% (*n* = 5)
Postgraduate degree	17% (*n* = 8)
Graduate Diploma/Graduate Certificate Advanced Diploma/Diploma	2% (*n* = 1)
Bachelor's degree	23% (*n* = 11)
Certificate I/II/III/IV	15% (*n* = 7)
High school	30% (*n* = 14)
Other	2% (*n* = 1)
**Employment**	
Employed full time	32% (*n* = 15)
Employment part time	13% (*n* = 6)
Self‐employed	9% (*n* = 4)
Casual employment	6% (*n* = 3)
Retired	36% (*n* = 17)
Homemaker	4% (*n* = 2)
**Current or previous experience working in healthcare**	
Yes	30% (*n* = 14)
No	70% (*n* = 33)
**Provided carer support to family member or friend**	
Yes	74% (*n* = 35)
No	26% (*n* = 12)

#### 3.1.7 Family Palliative Care Knowledge

Respondents' mean self‐rated knowledge of palliative care was slightly higher than mean self‐rated knowledge of end‐of‐life care. Comparisons of mean self‐rated knowledge and assessment of an individual's knowledge of palliative care through mean PaCKS scores are presented in Table 3.

#### 3.1.8 Family Experience and Familiarity With Palliative Care

The majority of respondents had previously heard of palliative care (96%, *n* = 45) and end‐of‐life care (85%, *n* = 40). Most participants agreed that they were familiar with palliative care (77%, *n* = 36) and end‐of‐life care (58%, *n* = 27). Just over half of respondents (53%, *n* = 25) agreed that they had experience with palliative care, slightly less (47%, *n* = 22) agreed that they had experience with end‐of‐life care.

#### 3.1.9 Within Group Comparisons

The mean *PaCKS* score was 9.7 (SD 3.1, range 3–13). There was no statistically significant difference in PaCKS scores based on gender (*p* = 0.1084), educational attainment (*p* = 0.2775), employment status (*p* = 0.6308), experience working in healthcare (*p* = 0.0932) or providing carer support (*p* = 0.3820). Comparisons between PaCKS score and responses to the items in the Experience and Familiarity with Palliative Care Questionnaire indicated that statistically significant differences in the mean *PaCKS* score were found when respondents reported being familiar with palliative care (*p* = 0.0273), having heard of end‐of‐life care (*p* = 0.0015), or having experience with end‐of‐life care (*p* = 0.0268). There were no statistically significant differences found between *PaCKS* scores and responses to the other items.

#### 3.1.10 Between Group Comparisons

There was no statistically significant difference between patient and family PaCKs scores (*p* = 0.6974). Examination of the PaCKs at item level highlighted knowledge deficits, including over half of participants not being aware palliative care can extend beyond the last 6 months of life (Table 7). Around a third of respondents did not know palliative care can address stress related to serious illness, improve a person's ability to participate in daily activities, or that patients could continue to see their usual doctors (Table 7).

**TABLE 7:** Percentage of participants with knowledge deficits per PaCKs item.

PaCKS item	Patient (*n* = 194)	Family (*n* = 47)
One goal of palliative care is to address any psychological issues brought up by serious illness.	31% (*n* = 61)	28% (*n* = 13)
Stress from serious illness can be addressed by palliative care	35% (*n* = 68)	36% (*n* = 17)
Palliative care can help people manage the side effects of their medical treatments	30% (*n* = 58)	23% (*n* = 11)
When people receive palliative care, they must give up their other doctors	37% (*n* = 71)	32% (*n* = 15)
Palliative care is exclusively for people who are in the last six months of life	51% (*n* = 98)	55% (*n* = 26)
Palliative care is specifically for people with cancer	19% (*n* = 36)	6% (*n* = 3)
People must be in the hospital to receive palliative care	25% (*n* = 48)	19% (*n* = 9)
Palliative care is designed specifically for older adults	16% (*n* = 32)	13% (*n* = 6)
Palliative care is a team‐based approach to care	20% (*n* = 38)	15% (*n* = 7)
One goal of palliative care is to help people better understand their treatment options	24% (*n* = 47)	26% (*n* = 12)
Palliative care encourages people to stop treatments aimed at curing their illness	28% (*n* = 54)	28% (*n* = 13)
One goal of palliative care is to improve a person's ability to participate in daily activities	37% (*n* = 72)	32% (*n* = 15)
Palliative care helps the whole family cope with a serious illness	14% (*n* = 27)	17% (*n* = 8)

*Note:* Knowledge deficit is based on an incorrect response as to whether an item was true or false, or if the respondent indicated they did not know if the statement was true or false.

### 3.2 Phase 2 Qualitative Findings

A total of 11 patients and nine family members participated in an interview. Purposive sampling was used to include participants who had attended or visited different wards and with varying levels of exposure to palliative care. Participant characteristics are outlined in Table 8 The interviews were conducted between April and June 2024. Interview duration ranged from 14 to 123 min, with a mean duration of 42 min. Three main themes and nine subthemes were identified from 79 codes generated from the analysis of the qualitative data (Figure 2). The three main themes were Communication, Knowledge and Perceptions, and Accessibility.

**TABLE 8:** Qualitative sample characteristics.

Variable	Patient sample (*n* = 11)	Family sample (*n* = 9)
Age mean (range)	64.7 (40–83)	59.4 (35–88)
**Gender**		
Woman	55% (*n* = 6)	56% (*n* = 5)
Man	45% (*n* = 5)	44% (*n* = 4)
**Education level**		
Postgraduate degree	18% (*n* = 2)	0% (*n* = 0)
Graduate diploma/Graduate certificate	18% (*n* = 2)	44% (*n* = 4)
Bachelor's degree	36% (*n* = 4)	33% (*n* = 3)
Certificate I/II/III/IV	9% (*n* = 1)	11% (*n* = 1)
High school	18% (*n* = 2)	11% (*n* = 1)
**Employment**		
Employed full time	9% (*n* = 1)	33% (*n* = 3)
Employment part time	9% (*n* = 1)	0% (*n* = 0)
Self‐employed	9% (*n* = 1)	11% (*n* = 1)
Casual employment	0% (*n* = 0)	11% (*n* = 1)
Retired	64% (*n* = 7)	44% (*n* = 4)
Unable to work	9% (*n* = 1)	0% (*n* = 0)
**Experience working in health care**		
Yes	27% (*n* = 3)	33% (*n* = 3)
No	73% (*n* = 8)	67% (*n* = 6)
**Provided carer support to family member or friend**		
Yes	36% (*n* = 4)	89% (*n* = 8)
No	64% (*n* = 7)	11% (*n* = 1)
**Palliative care experience or education**		
Yes	82% (*n* = 9)	89% (*n* = 8)
No	18% (*n* = 2)	11% (*n* = 1)
Mean PaCKs (SD)	11.45 (1.4)	10 (2.4)

**FIGURE 2:**
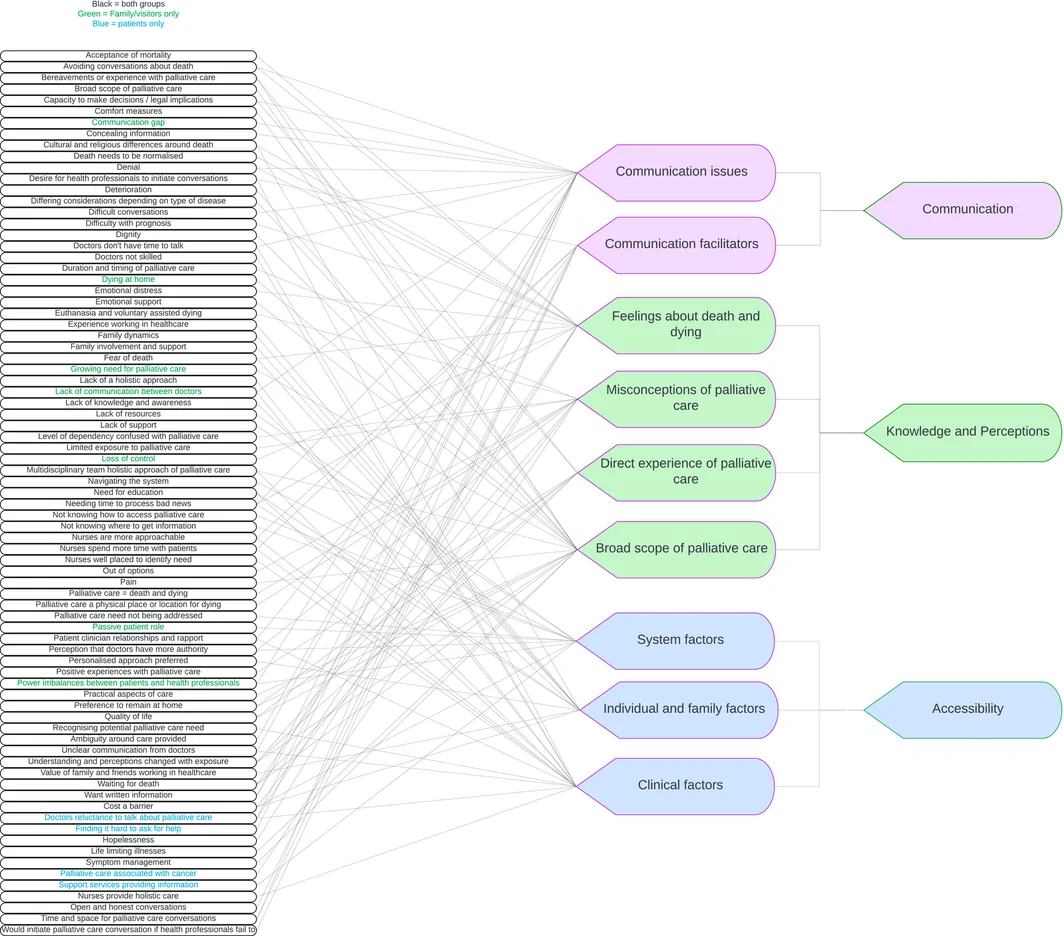
Thematic map. [Colour figure can be viewed at wileyonlinelibrary.com]

#### 3.2.1 Theme 1: Communication

This theme focuses on participants' experiences of discussing palliative care, including barriers and facilitators to effective conversations. While communication influenced participants' understanding of palliative care, the emphasis here is on *how* information was exchanged rather than the knowledge or beliefs themselves. Participants discussed issues that impeded communication, as well as facilitators that could promote communication related to palliative care. Some participants recounted negative experiences of having conversations about palliative care. Preferences on how information should be shared to better facilitate and improve communication were also raised.

##### 3.2.1.1 Communication Issues

Discomfort and fears associated with palliative care were identified as common issues that could prevent communication; ‘It's the fear of death… we don't want to talk about death’ (P6). Difficulty processing information about diagnosis and prognosis were viewed as a reason to delay conversations relating to palliative care; ‘…having a conversation like that is enough in itself, without touching on palliative care… people get such bad news, you don't want to start talking about palliative care right there and then’ (F2). Some participants recounted experiences where loved ones had concealed information about their prognosis and need for palliative care. This lack of communication was distressing and left family members unprepared; ‘We had no idea what we were in for… Dad lied to us’ (F9). Another participant recounted how the family and treating team concealed information from their loved one who was dying; ‘…we didn't really have the conversations with him in the room… I don't think we really told him that it was palliative care because that might be a bit scary…’ (P4). This concealment by the family aimed to protect their loved one.

The complexity and time needed to discuss palliative care were raised as barriers to having these conversations with doctors; ‘Our doctor, literally we get about two minutes of his time. So, I feel that's something that wouldn't be covered and I wouldn't be able to ask questions*’* (F3). There was also acknowledgment that palliative care conversations were difficult for all parties*:* ‘I think it's everyone who's involved… the patient… it's happening to them… their loved one, they don't want it to happen… doctors sometimes… get attached to patients and nurses as well…death is a very confronting thing’ (P6). Some participants reported issues with unclear communication about care plans and prognosis that resulted in confusion; ‘…from memory… I don't think they called it palliative care at the time… they just said, ‘We suggest you go into hospital… and have a break’ …I didn't realise he was going into palliative care… until I got there’ (P9). This lack of clear communication caused uncertainty and sometimes surprise when the topic of palliative care was eventually broached.

##### 3.2.1.2 Communication Facilitators

Participants expressed a desire for healthcare professionals to initiate palliative care conversations; ‘I think it should be fronted… straight up by the medical people’ (P11). Healthcare professionals having or building rapport with the patient was viewed as key to optimising communication; ‘…ideally… have a relationship with the person, so you're the person's doctor or nurse… It's harder if you come cold into the situation, but if you do come cold, you establish a relationship…’ (F5). Participants also wanted to be introduced to new people who could assist with palliative care by a healthcare professional they already knew and trusted; ‘…if someone would just approach me about palliative care… It wouldn't go down as well as if my oncologist was there…’ (P10).

Many participants felt nurses would be well suited to palliative care conversations*;* ‘…I think the nurses do an absolutely amazing job. They're the sort of people that I do trust, and I think coming from them, it would be quite good…’ (F8). The constant presence of nurses on the ward was in contrast to doctors who had very limited availability: ‘…they (doctors) rush in… and rush out… if you've got any questions… if you have a blank mind… you forget to ask… I'd say that nursing staff were probably the ones that you could best discuss that with…’ (P9). Open and honest conversations about palliative care were also viewed as key to facilitating meaningful communication ‘You can have an open conversation with me. Just hit me with it… and tell me’ (P5). Including clear information on the breadth of palliative care could also increase receptiveness to considering and discussing palliative care; ‘I think it's just really important for people to understand that it's not bound by time, and it is not bound by physical care like there is so much more that palliative care can guide them in…’ (F2).

Participants also expressed a desire for written information about palliative care to be provided in conjunction with other methods of communication: ‘…that people… can take that away with them and go home and talk about it and think about it. Something that's nicely presented… I think that can go a long way in helping’ (P11). Participants felt the addition of written information would allow an opportunity to understand what palliative care could offer, and would give them time to revisit and process information in their own time.

In summary, Theme 1 reveals that participants viewed communication as central to engagement with palliative care. Honest, timely discussions delivered by trusted clinicians, supported by written information, were considered essential for improving understanding and facilitating earlier conversations.

#### 3.2.2 Theme 2: Knowledge and Perceptions

This theme explores participants' understanding, beliefs and assumptions about palliative care. This theme focuses on the meanings participants attributed to palliative care and how these shaped their attitudes and expectations, whereas the Communication theme examines the process of information exchange. Feelings surrounding death and dying could impact perceptions of palliative care. Misconceptions regarding the scope and purpose of palliative care were common. Direct experience and exposure altered participants' perceptions of palliative care, and those with experience reported improved knowledge about the broad scope of palliative care.

##### 3.2.2.1 Feelings About Death and Dying

Participants identified how an individual's feelings about death and dying influenced their perceptions of palliative care. There were frequent associations made between death and dying, and negative emotions such as fear, hopelessness, and general emotional distress; ‘there would be devastation, a feeling of sadness, saying goodbye to your kids and hopelessness’ (P10). Participants explained that these feelings could result in denial and avoidance; ‘…the usual like just fear really and denial… so palliative care was not something I was prepared to even think about’ (F3). The negative emotions around death and dying were intensified in the context of premature death; ‘I think that the barriers are obviously it's emotional… it's pretty scary for people, like, if it's your daughter or your son in a younger situation, it's quite…. confronting’ (P11). Conversely, other participants described an acceptance of their mortality and that thinking about death did not cause them distress; ‘I'm quite realistic about my health… I could have a heart attack tomorrow… I know… I'm gonna die at some stage… so I wouldn't think it would be fear or hopelessness…’ (P5). Some participants felt that death needs to be normalised in society to reduce negative feelings associated with death and dying:

…we definitely have separated ourselves from death… It's… become a medicalised condition… death really is just a normal part of life… if we do… more culturally to normalise the fact… that it will happen… then things like talking [about] palliative care might not be so scary… (F4)

The feelings and emotions participants associated with death and dying influenced their perceptions of palliative care.

##### 3.2.2.2 Misconceptions of Palliative Care

The notion that palliative care was a physical place or location where patients were sent to die was frequently raised by participants; ‘Well, it's a place… like a hospital or type of hospital’ (P11). This was viewed as being separate from usual healthcare and some participants reported that they were not aware palliative care could be integrated in all healthcare settings; ‘I thought it was a specialised hospital or nursing home… I didn't realise… where we had mum and dad at that… I didn't realise they could do palliative care as well’ (F8). This strongly linked with the idea that palliative care was for the last days or weeks of life; ‘My understanding is that it's end‐of‐life care basically’ (P9). The misconception that palliative care was only for the end‐of‐life was recognised as a barrier to timely palliative care access; ‘I can imagine that people say, “Oh, you've said, I've got two years. I've got five years. Why are we talking about palliative care?”’ (F2).

Other participants reported that they associated palliative care with cancer; ‘…the first thing that I think of when I think of palliative care… is a serious cancer’ (P6). Some participants confused level of dependency with palliative care; ‘I think when they need 24‐hour care. They can't care for themselves anymore… they can't walk anymore, or they can't shower themselves’ (F2). Another participant raised concerns about financial costs of palliative care; ‘…another thing with the people of very low incomes might be… I don't understand the cost. Does it cost me? Can I afford it?’ (P11). Some participants also held misconceptions about which health professionals possess palliative care knowledge and specifically the role of nurses; ‘…doctors are better trained… have more information than nurses do… Nurses are there to take their blood pressure and they do all those sorts of things… they're not the doctor and the doctor is the one who knows’ (P6). This highlighted that participants who did not have experience with palliative care had misunderstandings or limited understanding of palliative care.

##### 3.2.2.3 Direct Experience of Palliative Care

Some participants reported on their own direct experiences with bereavements and palliative care. A few participants described how they were not aware that palliative care was being provided in the lead up to the death of their loved one. This was something that they had realised upon reflection; ‘So when I say he didn't actually get into palliative care, he did. But it was at his nursing home… they did both palliative care as well as nursing home… He passed pretty quickly…’ (F8). Similarly, another participant described being supported by community health professionals to care for his wife at home ‘…we kept her at home and I looked after her till she died… She died of a cancer… I suppose that's palliative care in the home’ (F5).

Participants who were prepared and understood that their loved one was approaching the end‐of‐life described their experience of waiting for death to occur; ‘…so I just might sound a bit weird, but I would try and practice being without him… because… we knew… there was no cure. It was only treatment… postponing everything’ (F7). Having clear and active palliative care involvement was described positively in participants experiences. Support received helped family members through challenging times; ‘…I think the whole experience… it wasn't fantastic, having your father pass away, but I think certainly the nursing staff… were actually really, really good… They made it quite easy’ (F8). Another participant identified that the early provision of palliative care enabled their loved one's wishes to be met and to die at home comfortably; ‘She just calmly was at home… she gradually deteriorated, but it was the way death should be but often isn't’ (F5).

In contrast when participants discussed very late integration of palliative care, they identified that this contributed to a reduced quality of life for their loved one; ‘…we have one of the family brought it up to two days before… at the end, when you sat and looked at it, she did not have any value left’ (P1). A lack of timely palliative care discussions led to ambiguity about the individual's wishes and rushed decisions while the person was immediately dying; ‘…I think… probably his wish was to stay at home, to be quite honest, we never really discussed that… Going into hospital at the last minute… We went in on the Wednesday, and he died on the Thursday’ (P9). This participant also described how her experience had changed her understanding of palliative care and when it should be introduced; ‘…if I had not gone through this with my husband… I'd be aware that palliative care is there, but I'm not sure whether I would discuss it. But having been in that situation… I would discuss it early’ (P9). These accounts from participants with direct experience indicated that they valued palliative care and understood the benefits of early integration.

##### 3.2.2.4 Broad Scope of Palliative Care

Some participants understood that palliative care could be integrated over extended periods of time; ‘Support of the patient and the family, through the last years of their life… or last months, or weeks and providing them with information and support for different things that they might need…’ (P7). Another participant described how completing a one‐day educational course improved their understanding of when palliative care could be introduced; ‘…realised then that palliative care is not just end‐of‐life. That it… can start quite a lot sooner and it's just more of a support and you can still be treated…it's much broader than…most people think…’ (F3). Symptom management and keeping patients comfortable were core elements of palliative care frequently mentioned by participants; ‘…my understanding of palliative care… We're trying to make the patient as comfortable as possible, good quality of life, pain control especially… they're vomiting, the proper medications and so forth…’ (P5).

The importance of individualised palliative care to cater for differing patient needs was also recognised ‘…it depends what the patient's condition is, what the prognosis is. All those things…’ (P5). Some participants were aware that specialist palliative care had a multidisciplinary team approach; ‘Palliative care teams are brought in for a kind of more holistic approach to end‐of‐life care’ (F4). There was also recognition that there is a growing demand for palliative care; ‘…it's a huge problem for Australia… that we have got and the longer we're living, of course, the problem is becoming bigger’ (F1). These responses demonstrate an understanding that the complex needs of an ageing population will require the integration of palliative care to optimise patient care.

In summary, Theme 2 describes how participants' knowledge and perceptions of palliative care varied considerably. Misconceptions commonly centred on palliative care being synonymous with imminent death, whereas direct experience was associated with a broader understanding of its supportive role throughout serious illness.

#### 3.2.3 Theme 3: Accessibility

Accessibility includes both barriers and facilitators to accessing palliative care services. This theme examines factors influencing accessibility, ranging from a broader healthcare system perspective through to the experiences and actions of individual patients, family members and healthcare professionals.

##### 3.2.3.1 System Factors

Difficulties with the general pressures and large demand placed upon healthcare services was raised by participants. This included the lack of resources within the healthcare system to meet this growing demand; ‘…the population has outgrown the service at the hospital and… unfortunately there's just not enough people there in the emergency and emergency is not big enough to handle the volume of people coming in’ (P1). There was also awareness that the disparities in services available across Western Australia could reduce palliative care accessibility for people living in regional and remote areas; ‘…you look at the location of the palliative care… if you lived in the back of [rural location] somewhere, there may not be something around there’ (P5).

A lack of support within busy and overwhelmed healthcare systems was also identified by some participants. One participant described the impacts of a system focused on physical treatment and a lack of holistic care and support;

the hardest thing it's been such a lonely journey… it's very, very sterile the [chemotherapy day unit]… even though you're right next to people… there's no scope for… interaction. I haven't… spoken to anyone else really apart from doctors and nurses about the treatment [husband's name] having… it's disappointing (F3)

This need for broader support within the healthcare system beyond providing treatment was echoed by another participant; ‘… needing some service through all of the medical system, not just the bit the specialist is concerned with treating’ (F4). Difficulties with navigating the system and not knowing what services were available were also raised by participants:

…unless you ask the right question, you don't always get the right answer… I went to the oncologist, and I made some comment… and he asked me to clarify it and once I got it out, he said “Oh look, just go to the doctor and get this… chronic disease management plan…” But I didn't know to ask, it just happened to come up and he recognised that there was a need for it… (P2)

This account highlights an element of luck or chance as to whether or not patients and their families would be connected with available services and supports. Another participant described actively seeking support but meeting obstacles when trying to access this; … I often was trying to get information and I just couldn't get it, or I didn't know who to ask, or I wasn't sure how to navigate the system (F4).

The hierarchal nature of healthcare could also be a barrier to access with some participants believing only a doctor could or should initiate palliative care; …the public would want information from a more authoritative person… I don't think… advice from a nurse would have sufficient weight (F1). Other participants expressed that they felt that within the healthcare system, the approach nurses adopted would make them better able to connect with patients about palliative care; …nurses sort of have a more holistic approach… I would probably feel more comfortable talking to a nurse because… it's more likely to come up in conversation, things that I need… (P2).

Compounding these issues with access in some cases, was the assumption of a passive patient role with patients entrusting all elements of their care to their treating physician. One participant described how her husband had adopted a passive role in his care ‘It was really interesting because my husband isn't old, he's 45. I would have thought the whole, “Doctor knows best”… is… on the way out with the old generation, but it made me realise… this still happens’ (F2). This had the potential to block access to supports and services that could be beneficial for patients, including palliative care ‘…I can imagine that if patients aren't told about this, they go “So I must not need it. There must be no need for it because they haven't mentioned it”’ (F2). Failures within the system to identify and facilitate access to palliative care led to suboptimal patient care; ‘they weren't getting the care that they deserved or asked for or what was expected…’ (P4). Participant responses highlighted an overall dissatisfaction with the healthcare system.

##### 3.2.3.2 Individual and Family Factors

The importance of recognising that time was needed to process life changing news and waiting for the person to be ready to discuss palliative care was highlighted as an important way to facilitate access; ‘…you need someone to either remind you or explain or… talk through things at a pace that you're comfortable with’ (P2). Family dynamics were frequently raised as a factor that could prevent planning and discussions that could facilitate access to palliative care. One participant described how her daughter's negative reaction to her treating doctor suggesting she should organise an advanced health care directive, had become a barrier to making plans about her future care needs and wishes; ‘I've just been putting it off… I don't really know why, but my daughter was upset… thought the doctor was saying I'm dying… so I'm reluctant… to bring up anything like that, because I feel as though it's too hard’ (P9). Another participant described a situation where there was an immediate palliative care need and refusal by family members to accept the situation had led to impasses when trying to access palliative care; ‘She has a son who is next of kin, but he's not responding. So, we have a problem there of where do we go to resolve that?’ (F1). Cultural and religious differences were also recognised as a factor that could influence individual and family perceptions of palliative care*;* ‘… maybe there's a religious restriction there somewhere’ (P5).

Some participants discussed being open to the idea of receiving palliative care but having a lack of knowledge about how or when palliative could be accessed; ‘If I had an understanding of when it could be accessed and who could access it and I've met the criteria, then I wouldn't personally have an issue with it…’ (P3). Participants with family and friends working in healthcare discussed how having someone with inside knowledge could help them manage their health and facilitate care; ‘I'm really lucky. I've got friends and relatives who are nurses and doctors… I often just ring them, and I say, “Do I need to worry about this symptom?” …I'm really grateful… I've got… other contacts’ (F3). Other participants described the need for better education for the general public to provide individuals with an understanding of palliative care and an awareness of what they could access; ‘Educating people, because if you know what palliative care means and what that encompasses, it might give you more idea of what's available’ (P2). Possessing this knowledge would give individuals and families the ability to ask questions about palliative care and could facilitate access.

##### 3.2.3.3 Clinical Factors

The unpredictable course of some diseases was raised as a clinical factor that could create uncertainty about when palliative care should be introduced; ‘…it's a really tough one… when do you bring this up… when do you start talking about palliative care when there isn't… a definitive? Saying (when) you will die’ (F2). Another participant described how information about the expected course of disease was withheld by the treating doctor and how this impeded the ability to plan:

I remember Mum coming and saying that they'd had a conversation with the doctor, but that was a long time afterwards and they said people with this usually don't live longer than a year. And it was like… that would have been handy to know… months ago (P2).

Another participant described disagreements between the oncologist and geriatrician on the clinical needs of their father:

…almost that they were fighting… I think it was happening though because of that difference in attitude… The oncologist as far as he was concerned… the treatment had worked… But the geriatrician was like, “Hmm, but there's this other stuff happening” …Basically my father died from the treatment, not the melanoma… it just felt disjointed (F4)

This was echoed by other participants descriptions of specialists who focused on one clinical issue rather than considering the bigger picture of what was happening for patients; ‘Surgeons tend to think about the surgery and the healing… I think they only thought of the surgical side. They never thought of the medical side of things…’ (P7).

The compartmentalisation of care was compounded by the approach many participants described having experienced where doctors did not actively engage with patients in clinical decision making; ‘Some doctors and specialists don't have a real great bedside manner unfortunately (laughs). They talk to people around them rather than the patient…’ (F1). This tendency was linked by some participants as many doctors not having the appropriate skill set to facilitate discussions about palliative care; ‘…I don't think doctors have those skills and my experience of doctors in hospitals… is that they just they deal with the facts. They don't get involved emotionally… it's just, “you know, this is what we're gonna do” …’ (P6).

Participants described how after a doctor had initially informed a patient about the need for palliative care, nurses were the clinicians that they perceived to be involved in the organisation and delivery of palliative care:

…the nurses are the ones that… set it all in motion… I think that's probably by far the best way to go about it because doctors, (a) they don't have time for it and (b) they're not the ones dealing with it all (F2)

The more frequent and prolonged contact patients and their families have with nurses in providing clinical care was also recognised as a factor that meant nurses could facilitate access to palliative care; ‘…as a patient, you're more familiar with the nurse. On a regular basis for a lot longer than you see the doctors…’ (P7). The ability for clinicians to empower patients when adopting a holistic person‐centred approach was highlighted in one participant's account;

…we went to radiation…one of the nurses there she asked me specifically, “What's most important to you?” …that was that was really cool because… I haven't been asked that before… when you go to a doctor, they say, “Well, we've got these results and this is our plan”…then they say, “Do you have any questions about that?” (P2).

This clinical approach actively engaged the patient in their care and provided an opportunity for broader discussions about the patient's wishes and care needs.

In summary, Theme 3 describes how participants' access to palliative care was influenced by interacting system, clinical and individual factors. Participants described barriers related to healthcare navigation, clinician practices and family dynamics, while emphasising that timely information, person‐centred care and improved public awareness could facilitate access.

### 3.3 Mixed Methods Integration

Quantitative results and qualitative findings were integrated via a joint display to identify mixed methods meta‐inferences with reference to the study objectives (Teddlie and Tashakkori 2009). This process identified four areas of expansion and two areas of confirmation (Data S1). Qualitative findings confirmed mixed responses about preferred terminology identified in the quantitative results and also confirmed the result that the majority of survey respondents would feel comforted by palliative care involvement. Areas of expansion included qualitative data explaining differing perceptions of palliative care, factors that influenced understanding of palliative care, and identification and understanding of the barriers to palliative care.

## 4 Discussion

The aim of this study was to identify barriers to timely access to palliative care from the perspective of Australian inpatients and their families. The findings of this study revealed a wide variety of complex issues that could impede timely access to palliative care from the perspective of health consumers. Barriers were identified at the individual, health professional and system level.

Similar to research exploring health consumer perspectives in other healthcare contexts, misconceptions of palliative care and lack of knowledge featured strongly in the quantitative results and qualitative findings (Slusser et al. 2024). Further exploration of knowledge deficits identified in patient and family surveys in the qualitative interviews provided a deeper understanding of these results. Views of palliative care being for the immediate end‐of‐life, a place or location where patients were sent to die, or only for cancer patients highlighted a lack of understanding about palliative care.

Mirroring the quantitative results, where participants who self‐reported higher levels of experience and familiarity with palliative care and end‐of‐life care had statistically significant higher PaCKs scores, participants described how their understanding of palliative care changed with direct experience. Those with direct experience described these positively, and demonstrated an understanding of the broad scope of palliative care and the supports that could be provided. Gaps in knowledge and misconceptions of palliative care highlight the need for palliative care education for the general public and health professionals to ensure individuals are empowered and aware of the supports available to people living with life‐limiting illnesses (Li et al. 2021).

Issues with poor communication, lack of time to communicate, avoidance of difficult conversations by health professionals, patients, and family featured strongly in the qualitative findings. A lack of skills and knowledge in health professionals regarding palliative care and how to clearly communicate on this topic was also raised, reflecting the lack of education and training to facilitate generalist palliative care in practice (Klaiman et al. 2024; WHO 2024). In this study, nurses were generally seen as the preferred profession to lead palliative care due to being perceived as more available and approachable to patients and their families. This suggests nurse‐led approaches could better facilitate palliative care and reflects the holistic nursing approach and origins of modern palliative care as a nurse‐led movement (Graham and Clark 2008).

Interestingly, differing communication difficulties across patient groups including those who are culturally and linguistically diverse did not feature in this study, likely due to the private metropolitan setting. In other healthcare settings this could present a further barrier to communication (Slusser et al. 2024). Patient experiences will vary based on healthcare context and individual factors, and these must be considered when assessing the transferability of qualitative findings.

The mixed findings in relation to terminology including palliative, supportive, and end‐of‐life care in both the quantitative and qualitative data highlight the difficulties in how to effectively communicate. Given the negative connotations associated with the terms ‘palliative and end‐of‐life care’ the adoption of softer terminology such as ‘supportive care’ has been implemented and advocated in some areas (Taylor and Davies 2025). The very mixed responses in our study point to there being no easy fix via a simple name change. If the underlying lack of knowledge and misconceptions are not addressed this could result in any alternative name being similarly misunderstood in the future. As highlighted in our study and the wider research, societal attitudes towards death and dying also influence individuals' feelings about palliative care with fears of death and dying impeding communication about and facilitation of palliative care (Huang et al. 2022; Ruíz‐Fernández et al. 2021).

An acute awareness of the challenges faced in healthcare and the barriers these present to palliative care access was identified in the qualitative data. These reflect issues identified in international studies including insufficient staffing (Klaiman et al. 2024; Rost et al. 2020) and the huge demands being placed on healthcare systems in the context of ageing populations (Buchan and Catton 2023). The increasing number of older patients with chronic and complex healthcare needs is creating a huge need for palliative care. The current lack of generalist palliative skills and training has led to specialist palliative care services being inundated with referrals often with little or no increase in resources to cope with this large demand (Palliative Care Australia 2025). Specific to the Australian healthcare context, participants also discussed issues about the provision of palliative care across vast and sparsely populated states such as Western Australia. Disparities in healthcare experienced by those living in remote and regional areas in Australia (AIHW 2024b) are a further barrier to accessing palliative care.

### 4.1 Strengths and Limitations

This study provides further understanding of the perspective of health consumers on the barriers to palliative care, a previously underreported area. Utilising a mixed methods research approach allowed for deeper understanding of participants' level of palliative care knowledge and the complex issues that can impede palliative care access. The main limitation of the study is that it was conducted in a single healthcare setting which limits the generalisability of the quantitative results and transferability of the qualitative findings.

### 4.2 Recommendations for Future Research

Targeted interventions need to be co‐designed, implemented and trialled with health consumers and clinicians in future research to address the issues identified and facilitate timely access to palliative care. These could include testing targeted communication strategies and educational interventions to improve understanding and overcome misconceptions of palliative care at a public and health professional level.

### 4.3 Implications for Policy and Practice

- Government initiatives are needed to prioritise palliative care and ensure the optimal care of ageing populations,
- Public health campaigns are needed to improve health literacy and understanding of palliative care to overcome barriers related to knowledge and perceptions,
- Adequate funding is required to improve palliative care access particularly for rural and remote communities,
- Education on palliative care should be mandated for all health professionals to address the gaps at the health professional level identified by health consumers,
- A focus on identifying palliative care need and how to communicate with patients and families about palliative care is needed in health professional training,
- Collaboration and multidisciplinary practice should be fostered to facilitate early access to palliative care to optimise patient care and outcomes.

## 5 Conclusion

This study provides further insight into the barriers to palliative care from the perspective of health consumers, a previously under‐reported area. Patients and family members who participated in the study perceived a range of barriers to palliative care at the individual, health professional and system level that included communication, knowledge and perceptions, and accessibility. These findings highlight areas where targeted interventions should be developed to improve timely access to palliative care.

## Funding

The completion of this mixed methods study was supported as part of an AUD$50,000 Seed Funding Grant awarded to ALC from the St John of God Foundation.

## Disclosure

*Statistics*: There is a statistician on the author team; the author is Huaqiong Zhou.

## Conflicts of Interest

The authors declare no conflicts of interest.

## Supporting information


**Data S1:** Joint display.

**Data S2:** Good reporting of a mixed methods study (GRAMMS) checklist.

## Data Availability

The data that support the findings of this study are available on request from the corresponding author. The data are not publicly available due to privacy or ethical restrictions.
