# Supplementary material for: Consumer Perspectives on the Barriers to Timely Palliative Care Access: A Mixed Methods Study
Source: J Clin Nurs. 2026 Aug 5;35(12):5442–57. doi: 10.1111/jocn.70480 (PMC13744240; doi:10.1111/jocn.70480)
Supplement: Supplementary file 1 [file JOCN-35-5442-s001.docx]

**Supplementary File 1. Joint Display**

| **Study objective** | **Quantitative results** | **Qualitative findings** | **Mixed methods meta-inferences** |
| --- | --- | --- | --- |
| Explore perceptions of palliative care from patient and family perspectives | Patients: Majority would feel comforted if palliative care was involved (68%, n=133).  Patients: Mixed responses about feeling frightened or hopeless if referred to palliative care.  Patients: Mixed responses about terminology supportive care vs palliative care  Patients and family: Higher self-rated knowledge of palliative care compared to end of life care | **Subtheme: Feelings about death and dying**   - Frequent associations with death and negative emotions such as fear and hopelessness, - Individual feelings about death and dying influenced perceptions of palliative care.   **Subtheme: Misconceptions of palliative care**   - A place or location to die, - Confusion with level of dependency, - Concerns around cost, - For cancer patients,   **Subtheme: Direct experience of palliative care**   - Being prepared, - Having palliative care involvement was described positively, - Exposure changed perceptions of palliative care, - Enabled wishes to be met.   **Subtheme: Broad scope of palliative**   - When palliative care can be introduced, - Extended time palliative care can be provided for, - Symptom management and keeping comfortable, - MDT approach. | **Confirmation**: Qualitative findings around direct experience of palliative care confirmed quantitative results where the majority of patients reported they would feel comforted by palliative care involvement  **Expansion**: Qualitative data explained the reasons people may feel frightened or hopeless and why some people did not have concerns about palliative care or death and dying.  **Confirmation**: Participants expressed a range of opinions regarding terminology reflecting and confirming the mixed responses in the quantitative data  **Expansion**: Level of understanding of palliative care and end of life care and the factors that influenced this revealed in the qualitative data |
| Identify barriers to generalist palliative care approaches and specialist palliative care referral | Patient: The majority did not have experience of palliative care (61%, n=118) or end of life care (69%, n=134)  Family: Around half of family respondents reported having experience of palliative care (53%, n=25) and end of life care (47%, n=22) | **Subtheme: Communication Issues**   - Avoiding conversations about death and dying, - Time needed to process, - Lack of time and information.   **Subtheme: System factors**   - Lack of resources, - Growing demand on health services, - Inequities in healthcare, - Lack of holistic care, - Hierarchical nature of healthcare.   **Subtheme: Individual and family factors**   - Time needed to process bad news, - Family dynamics, - Denial, - Need for education.   **Subtheme: Clinical factors**   - Unpredictable course of disease, - Professionals withholding information from patients, - Conflict in MDT, - Lack of holistic care, - Failure to identify palliative care need, - Lack of skills and knowledge of health professionals. | **Expansion**: A wide range of barriers to palliative care were identified in the qualitative data expanding on the quantitative results that indicated limited experience of palliative and end of life care. |
| Understand the barriers to generalist palliative care approaches and specialist palliative care referral | Patient/family: Almost half of respondents believed palliative care was only for last six months of life  Patient/family: Around a third of respondents thought they would have to give up their other doctors if referred to palliative care  Patient/family: Around a third of respondents thought palliative care could stress from serious illness could not be addressed by palliative care  Patient/family: Around a third of respondents didn’t realise a goal of care is improve a person’s ability to participate in daily activities  Statistically significant higher PaCKS scores when family and patient respondents reported being familiar or having experience with palliative care or end of life care. | **Subtheme: Communication Issues**   - Discomfort and fear could prevent communication, - Complexity and time needed for palliative care discussions.   **Subtheme: Misconceptions of palliative care**   - Last few days or weeks of life   **Subtheme: Direct experience of Palliative care**   - Unaware that palliative care was being provided, - Late or untimely access to palliative care led to poor quality of life/ death.   **Subtheme: System factors**   - Difficulty navigating the system   **Subtheme: Individual and family factors**   - Not knowing how or when to access palliative care   **Subtheme: Clinical factors**   - Fragmented care | **Expansion**: The qualitative data provided an understanding of barriers identified in the quantitative results and revealed other explanations for other factors that could be barriers. |
