# Supplementary material for: Consumer Perspectives on the Barriers to Timely Palliative Care Access: A Mixed Methods Study
Source: J Clin Nurs. 2026 Aug 5;35(12):5442–57. doi: 10.1111/jocn.70480 (PMC13744240; doi:10.1111/jocn.70480)
Supplement: Supplementary file 2 [file JOCN-35-5442-s002.docx]

**Good Reporting of A Mixed Methods Study (GRAMMS) checklist**

| **Item** | **Reported** |
| --- | --- |
| Describe the justification for using a mixed methods approach to the research question | Design, page 4-5. |
| Describe the design in terms of the purpose, priority and sequence of methods | Design, page 4-5 and Figure 1 |
| Describe each method in terms of sampling, data collection and analysis | Quantitative methods page 5-7  Qualitative methods page 7-8 and Table 1 |
| Describe where integration has occurred, how it has occurred and who has participated in it | Design page 4-5  Mixed Integration page 8 and 22 |
| Describe any limitation of one method associated with the present of the other method | Strengths and limitations, page 24 |
| Describe any insights gained from mixing or integrating methods | Discussion, page 22-25 |

Reference: O'Cathain, A., Murphy, E., & Nicholl, J. (2008). The quality of mixed methods studies in health services research. *Journal of Health Services Research & Policy*, 13(2), 92-98. <https://doi.org/10.1258/jhsrp.2007.007074>
